# Changes in Internalizing Symptoms During the COVID-19 Pandemic in a Transdiagnostic Sample of Youth: Exploring Mediators and Predictors

**DOI:** 10.1007/s10578-022-01382-z

**Published:** 2022-07-06

**Authors:** Simone P. Haller, Camille Archer, Annie Jeong, Allison Jaffe, Emily L. Jones, Anita Harrewijn, Reut Naim, Julia O. Linke, Joel Stoddard, Melissa A. Brotman

**Affiliations:** 1 Emotion and Development Branch, National Institutes of Mental Health, 9000 Rockville Pike, Bldg. 15K, Bethesda, MD 20892-2670 USA; 2 Department of Psychology, Education and Child Studies, Erasmus University Rotterdam, Rotterdam, The Netherlands; 3 Department of Psychiatry & Neuroscience Program, Children’s Hospital Colorado, Pediatric Mental Health Institute, University of Colorado, Anschutz Medical Campus, 13123 East 16th Avenue, Aurora, CO 80045 USA

**Keywords:** Stress, Anxiety, Depression, Children and adolescence, fMRI, Threat bias

## Abstract

The COVID-19 pandemic is a chronically stressful event, particularly for youth. Here, we examine (i) changes in mood and anxiety symtpoms, (ii) pandemic-related stress as a mediator of change in symptoms, and (ii) threat processing biases as a predictor of increased anxiety during the pandemic. A clinically well-characterized sample of 81 youth ages 8–18 years (*M* = 13.8 years, *SD* = 2.65; 40.7% female) including youth with affective and/or behavioral psychiatric diagnoses and youth without psychopathology completed pre- and during pandemic assessments of anxiety and depression and COVID-related stress. Forty-six youth also completed a threat processing fMRI task pre-pandemic. Anxiety and depression significantly increased during the pandemic (all *p*s < 0.05). Significant symptom change was partially mediated by pandemic stress and worries. Increased prefrontal activity in response to neutral faces pre-pandemic was associated with more intense parent-reported anxiety during the pandemic (all *Fs*(1.95,81.86) > 14.44, *ps* < 0.001). The present work extends existing knowledge on the mediating role of psychological stress on symptoms of anxiety and depression in youth.

**Supplementary Information:**

The online version contains supplementary material available at 10.1007/s10578-022-01382-z.

## Introduction

The COVID-19 pandemic has had great impact on the lives of children and adolescents, for instance, over 140,000 children under age 18 in the U.S. [1] and over 1.1 M globally have lost a parent or custodial grandparent to COVID-19 [2]. Beyond fears of viral infection and death, many families report economic and educational setbacks, and ongoing psychosocial challenges related also to mitigation efforts including school closures and social distancing measures [3, 4]. Several studies have revealed the clinical impact of the pandemic, with increased levels of mood and anxiety symptoms reported in youth and young adults [3, 5–9]. A recent meta-analysis reported global prevalence estimates of child and adolescent depression and anxiety to have doubled during the pandemic with estimates of ~ 25% and 21% respectively [10]. However, few studies have employed prospective designs that include clinical or neurocognitive assessments prior to the onset of the pandemic [11]. A longitudinal design provides a unique opportunity to inform our understanding of how the COVID-19 pandemic and associated public health measures have impacted youth. Specifically, we can examine how pre-existing variability in clinical and neural indicators may exacerbate stress responses and risk for internalizing symptoms [12]. Understanding which factors leave youth more susceptible to stress may help identify individuals at risk for anxiety early and support work on preventative strategies.

Late childhood and in particular adolescence as a developmental period is characterized by increased independence, continued development in emotion regulation, heightened parent-youth conflict, and emphasis on peer relationships for well-being and social learning [13]. It is also often the time of first onset of mood and anxiety disorders [14]. Hence, psychosocial stressors may be particularly impactful during this period, with potentially long-term consequences [15]. Reports on the impact of pandemic-related stress on youth provide some evidence that anxiety and depression symptoms track with perceived pandemic-related stress levels [16–19] and that pre-pandemic anxiety levels predict increases in anxiety during the pandemic [3, 20–22]. However, findings are inconsistent and also include reports of stability and reductions in mental health difficulties such as anxiety [3, 23–27], possibly due to the removal of some daily stressors (e.g., school, social interactions). These discrepancies highlight the need to better understand which youth are at high risk for stress-related increases in symptoms.

In addition to pre-existing symptoms, neurocognitive factors may impact stress reactivity [28]. Cognitive models of anxiety highlight the role of attention, interpretation and memory for negative affective information in the development and maintenance of symptoms [29]. Previous work has established links between anxiety and hypervigilance to threat and biased interpretations [13, 30, 31]. Neuroimaging work has established both cross-sectional and longitudinal associations between anxiety symptoms ventral and lateral prefrontal-cortex activation while attending to and appraising threatening information [32, 33]. Additionally, prospective relationships between amygdala reactivity to negative face-emotions and internalizing outcomes for those who experienced stressful life events have been reported [34]. Attention allocation to social threats (e.g., angry faces) or biased interpretations of ambiguous social cues (e.g., neutral faces) may predispose youth to experience social situations during the pandemic as more stressful [35, 36]. Early evidence suggests prospective associations between activation patterns to face-emotions and internalizing symptoms during the COVID-19 pandemic. Weissman and colleagues [37] found that increased amygdala activation to neutral faces relative to fearful faces pre-pandemic predicted internalizing outcomes during the pandemic. Other work in adult volunteers has found increased activation in the anterior insular to uncertain threat to predict increased COVID-related negative affect [38]. Thus, tentatively, the functioning of the amygdala-prefrontal cortex regulatory network during the presentation of potentially threating social cues may be associated with anxiety levels during the pandemic.

### The Present Study

Here, we longitudinally assessed a transdiagnostic sample of youth with varying levels of affective psychopathology during the first ~ 11 months of the pandemic. The objectives of the study were three-fold. First, we examined changes in anxiety and depression levels from pre- to during-pandemic in youth with pre-existing affective psychopathology and healthy control youth. Second, we tested pandemic-related stress as a mediator of increases in anxiety and depression. Third, in a subsample, we also investigated whether attention towards threat and associated whole-brain activation patterns assessed pre-pandemic were associated with increases in anxiety during the pandemic.

### Hypotheses

Consistent with previous work, we hypothesized increases in anxiety and depression, partially mediated by reports of pandemic-related stress and worries. We also expected that hypervigilance to threat prior to the pandemic and associated activation patterns in regulatory and salience circuitry (e.g., anterior insular and cingulate cortex and dorso-lateral prefrontal cortex) would predict increases in anxiety during the pandemic.

## Materials and Methods

### Participants

One-hundred and fifty-one youth had been enrolled in phenotyping and treatment protocols at the National Institute of Mental Health (NIMH) within ~ 2 years prior to 3/16/2020 (day of school closures in Maryland) and had completed an fMRI threat processing scan or participated in other behavioral tasks pre-pandemic. To be enrolled in these protocols, participants had to be aged 8 to 18 years and meet criteria for a primary diagnosis of Disruptive Mood Dysregulation Disorder (DMDD), Attention Deficit/Hyperactivity Disorder (ADHD) or an anxiety disorder (generalized, social, and/or separation anxiety disorder). Youth with no psychopathology were also enrolled. Diagnoses were determined via the lifetime version of the Kiddie Schedule for Affective Disorders and Schizophrenia [K-SADS-PL; 39], with a separate module for DMDD, by masters- or doctoral level clinicians at a comprehensive pre-study evaluation. Diagnoses were confirmed in regular consensus conferences chaired by a senior psychiatrist or clinical psychologist. Exclusion criteria comprised bipolar, psychotic, pervasive developmental, posttraumatic stress, and substance use disorders within the last 3 months or an IQ < 70 [40].

From the 151 youth identified for a during-pandemic follow-up assessment, 47 were no longer eligible to be contacted (age > 18 years) and 23 were not interested/able to participate. The final sample comprised eighty-one (81) youth (Age: *M* = 13.8 years, *SD* = 2.65; 40.7% female; see Table 1). Forty-seven participants (58%) had diagnoses of anxiety disorders, ADHD, Oppositional-Defiant Disorder (ODD) or DMDD. Thirty-four youth (42%) had no psychiatric diagnosis.

**Table 1:** Participant characteristics for the full sample (*N* = 81) and the subsample that completed the threat fMRI task (*N* = 46)

Characteristic	N = 81
Age M(SD)	13.84 (2.65)
Sex *n*(% Female)	33 (41)
Race *n*(%)	
American Indian or Alaska Native	1 (1.2)
Asian	2 (2.5)
Black or African American	6 (7.4)
Multiple Races	11 (14)
White	58 (72)
Not reported	3 (3.7)
Ethnicity *n*(%)	
Latino or Hispanic	9 (11)
Not Latino or Hispanic	69 (85)
Not reported	3 (3.7)
Income *n*(%)	
Under $5,000	0 (0)
$5,000–$9,999	0 (0)
$10,000–$14,999	0 (0)
$15,000–$24,999	2 (2.5)
$25,000–$39,999	2 (2.5)
$40,000–$59,999	0 (0)
$60,000–$89,999	5 (6.2)
$90,000–$179,999	27 (33)
Over $180,000	25 (31)
Not reported	20 (25)
IQ M(SD)	113 (13)
Not reported	8
Diagnoses (based on K-SADS) *n*(%)	
DMDD	7 (8.6)
ADHD	27 (33)
Any Anxiety Disorder	29 (36)
ODD	13 (16)
MDD	0 (0)
No Diagnosis	34 (42)

Characteristic	*n* = 46
Diagnoses (based on K-SADS) *n*(%)	
DMDD	7 (15)
ADHD	8 (17)
Any Anxiety Disorder	14 (30)
ODD	7 (15)
MDD	0 (0)
No Diagnosis	14 (30)

*Note* IQ: Intelligence quotient as assessed by the WASI [40]

*ADHD* attention-deficit/hyperactivity disorder, *DMDD* disruptive mood dysregulation disorder; *MDD* Major Depressive Disorder, *ODD* oppositional defiant disorder (ODD)

A subset of 56 youths also underwent functional magnetic resonance imaging (fMRI) pre-pandemic, while completing a threat processing task, 46 youth generated usable data (age: *M* = 13.0 years, *SD* = 2.65, range = 8–18; 43.5% female, see Table 1).

Parents provided written informed consent and youth provided assent. Participants received compensation for their participation. Recruitment materials included direct mailings and online advertisements. All procedures were approved by the NIMH Institutional Review Board and in accordance with the Helsinki declaration and its later amendments.

### Measures

#### Clinician-Rated Screening Measure

##### Schedule for Affective Disorders and Schizophrenia for School-Age Children-Present and Lifetime Version

The Schedule for Affective Disorders and Schizophrenia for School-Age Children-Present and Lifetime Version (K-SADS-PL [39]) is an 82-symptom diagnostic semi-structured interview that probes present and lifetime symptoms of affective, anxiety, and externalizing disorders, as well asschizophrenia in children. Symptoms are rated on a 0–3 point scale. The K-SADS-PL has excellent interrater reliability (93% -100%) and fair to excellent test–retest reliability (κ = 0.63–1.00). The K-SADS-PL was administered by a graduate-level (doctoral or masters) clinician trained to reliability (κ > 0.7).

#### Parent- and Child-Report Symptom Measures

##### Short Mood and Feelings Questionnaire

The Short Mood and Feelings Questionnaire (SMFQ) is a 13-item measure of depressive symptoms in children over the last two weeks with both a parent (SMFQ-P) and child (SMFQ-C) report. The total score ranges from 0 to 26. The SMFQ-C has good internal consistency (α = 0.88 to 0.89) [41]. A cut-off score of 12 suggests clinically relevant depressive symptoms [41]. In our sample, the measure had good internal consistency (SMFQ-C: α = 0.91, SMFQ-P: α = 0.91) at the baseline assessment.

##### Screen for Child Anxiety Related Emotional Disorders

The Screen for Child Anxiety Related Emotional Disorders (SCARED) is a 38-item dual-informant questionnaire (parent: SCARED-P, child: SCARED-C) that surveys symptoms of anxiety disorders experienced over the past 3 months [42]. The total score ranges from 0 to 82. For both SCARED-C and SCARED-P a cutoff score of 25 or above has been suggested to indicate clinically significant anxiety [43]. The SCARED has good internal consistency (α = 0.74 to 0.93) and moderate to good test–retest reliability (Child: ICC = 0.59–0.61, Parent: ICCs = 0.74–0.86) [44]. In our sample, the measure had excellent internal consistency (SCARED-C: α = 0.94, SCARED-P: α = 0.92) at the baseline assessment.

Measure descriptions and results of two other important and sample-relevant dimensions of developmental psychopathology, irritability, and ADHD symptoms (specifically inattention and hyperactivity) are included in the supplements.

### Stress Measures

#### Coronavirus Impact Scale (CIS)

The Coronavirus Impact Scale is a 12-item parent-reported questionnaire assessing impact of the COVID-19 pandemic including access to food and health care, social support, employment status and routines. Scores range from 0 to 24; the total score is calculated by summing all multiple-choice items. Items have acceptable internal consistency (α = 0.64 to 0.75) [45]. In our sample, internal consistency was adequate with α = 0.77. Data from this sample was used to validate the scale in a separate publication [45].

#### Coronavirus Health Impact Survey (CRISIS)—COVID-19 Worries Subscale

The Coronavirus Health Impact Survey (CRISIS) is an 84-item questionnaire that assesses different domains relevant to stress vulnerability and resilience during the pandemic [46]. Five items specifically assess worries around COVID-19 infection and physical health; we derive a CRISIS worries score by summing these five items on the parent-reported form. In our sample, this subscale had good internal consistency (α = 0.83).

In addition to clinical and COVID stress measures, participants provided demographic information and information on household income.

### Statistical Analysis

Analyses were conducted using R v4.0. The alpha level was set at 0.05. Given prior reports of substantial informant discrepancies in the employed clinical measures [47, 48], we examined parent- and youth-reports separately. We conducted pairwise t-tests to examine change in each clinical measure from pre- to during the pandemic. Cohen’s *d* is computed as a measure of effect size, with *d* = 0.2 considered a small effect, *d* = 0.5 a medium and *d* = 0.8 a large effect [49]. Supplementary Materials contain an additional analyses examining change in symptoms separately for heathy controls and youth with pre-existing psychiatric diagnoses. For symptom domains that showed significant change, we examined pandemic-related stress and worries as a mediator of change. We fit multiple path analysis models using the R package lavaan [50] with bias-corrected and accelerated bootstrap intervals (BCa; 1000 sample iterations). Mediation models included the direct effect of pre-pandemic clinical measures on during-pandemic clinical measures and the indirect effect of pandemic-related stress (CIS, CRISIS). We examined pandemic stress as a mediator for significant change in three clinical measures for a total of six mediation models.

## fMRI Data

### fMRI Task

A subset of participants completed a canonical, fMRI-adapted threat attention task, the dot-probe task [51], pre-pandemic onset. During the task, a fixation cross (500 ms) preceded vertically paired faces (1500 ms) with either angry-neutral or neutral–neutral expressions followed by an arrow (500 ms; see Supplementary Fig. S1). Participants had to indicate the direction of the arrow, which appeared either behind the angry or the neutral face. Trials where the arrow appears behind the angry face are considered threat-congruent trials (i.e., participants attending to the threat will be quick to respond to the arrow), while trials where the arrow appears behind the neutral face are considered threat-incongruent trials (i.e., participants attending to threat will need to shift their attention away from the threat and respond more slowly). Neutral–neutral face pairings provided a non-threat condition (neutral trials). Task conditions were presented at random, with a jittered inter-trial interval (250 ms-750 ms), across two runs of 80 trials per trial type, interspersed with 80 fixation-only trials.

### Clinical Measures and Behavioral Measures

In the subsample of 46 youth with usable fMRI data, we examined whether parent- and child-reported measures significantly changed from pre- to during-pandemic using pairwise t-tests. Using reaction times, we calculated attention bias (incongruent—congruent trials), and threat bias (threat—neutral trials). We examined associations between these indices and during-pandemic anxiety, controlling for pre-pandemic anxiety and age.

### fMRI Data Acquisition and Processing

Neuroimaging data were collected on 3 T General Electric Signa 750 scanners using a 32-channel head coil. Blood-oxygen-level-dependent (BOLD) signal was measured by T2*-weighted echoplanar imaging at a voxel resolution of 2.5 × 2.5 × 3.0 mm (repetition time = 2300 ms, echo time = 25 ms, flip angle = 50°, field of view = 240mm^2^, frequency x phase: 96 × 96). A structural magnetization-prepared rapid gradient echo scan (MPRAGE; echo time/inversion time = minimum full echo time/725 ms; field of view = 220 mm^2^, frequency x phase = 256 × 192, 1 mm isotropic voxels) was acquired for co-registration with the functional data.

Data were analyzed using Analysis of Functional NeuroImages [AFNI; http://afni.nimh.nih.gov/afni/; 52] v21.0.08 using standard preprocessing (see Supplementary Materials). A general linear model estimated BOLD signal change for all three trial types (congruent, incongruent, neutral) and error trials. Two multivariate models [AFNI’s 3dMVM; 53] were computed for child- and parent-reported SCARED scores separately to examine associations between change in anxiety with the pandemic and brain activation to different task conditions. Specifically, we entered during-pandemic SCARED scores as a continuous variable, activation coefficients (congruent, incongruent, neutral) as the within-subjects variable and, to control for pre-pandemic anxiety, we entered pre-pandemic SCARED scores. Age was entered as a continuous covariate. Continuous variables (SCARED scores, age) were grand-mean centered. The main interaction of interest is the two-way condition-by-during-pandemic SCARED scores interaction.

To correct for multiple comparisons, Monte-Carlo simulations were performed using AFNI’s 3dClustSim with smoothness of the residuals estimated based on a Gaussian plus mono-exponential spatial autocorrelation function. Analyses were restricted to a gray matter mask of 84,750 voxels where 90% of participants had useable data. Across participants, the effective smoothness was FWHM = 9.27 mm (ACF parameters, a = 0.58, b = 3.40, c = 10.60). Two-sided thresholding was examined with first-nearest neighbor clustering; results were thresholded at a voxel-wise *p* < 0.005 and a cluster extent of *k* = 46 voxels to obtain a whole-brain family-wise error correction of *p* < 0.05. For post-hoc analyses and visualization in R version 4.0, average activity was extracted from each cluster. Thirty-six (36) participants’ data is also contained in another report [54].

### Procedure

After enrollment into phenotyping and/or treatment protocols, on a separate visit or online, via an established NIH online survey system, parents and children completed the symptom scales. Participants willing and able to scan completed an fMRI threat processing scan in a follow-up visit.

**Fig. 1:**
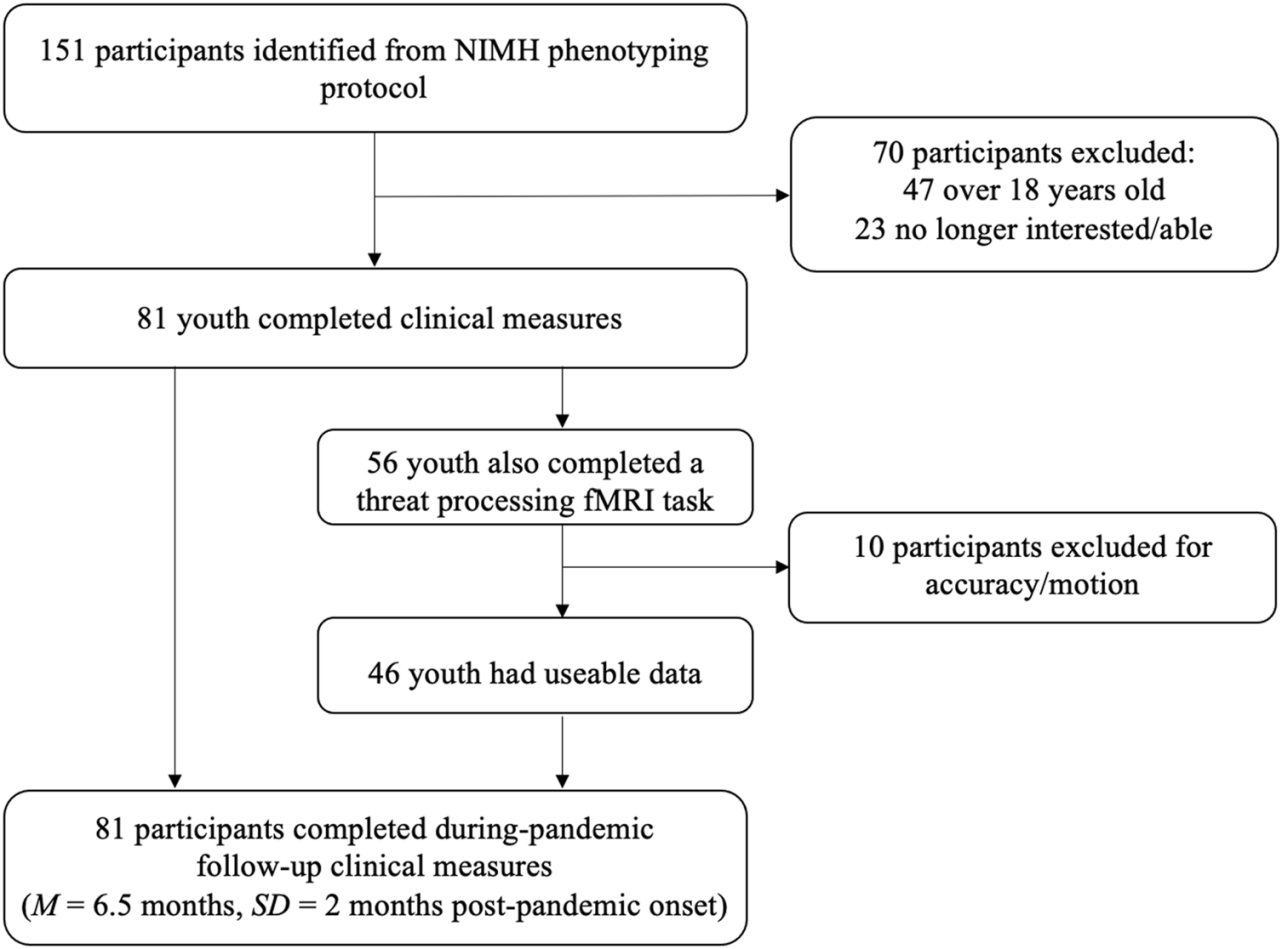
Schema detailing the flow of participants through the study

During the pandemic, all parents and children completed symptom measures through the NIH online survey system. The time interval between pre- and during-pandemic measures was *M* = 411.22 days/1.1 years, *SD* = 207.26 days/6.9 months, i.e., measures were taken *M* = 217 days/7.2 months, *SD* = 186 days/~ 6.2 months pre-pandemic and *M* = 195 days/6.5 month, *SD* = 61 days/2 months into the pandemic. Pre-pandemic parent- and child-report measures were completed within three months of the fMRI scan for the subsample that was included in the fMRI analysis (see Fig. 1).

## Results

### Change in Anxiety and Depression Levels

We observed significant increases during the pandemic in youth-reported depression (MFQ-C: *t*(72) = 2.64, *p* = 0.01, *d* = 0.31), as well as youth- and parent-reported anxiety (SCARED-C: *t*(74) = 3.09, *p* = 0.003, *d* = 0.36; SCARED-P: *t*(72) = 2.33, *p* = 0.02, *d* = 0.28). No significant changes were found in parent-reported depression (MFQ-P: (*t*(67) = 1.59, *p* = 0.12, *d* = 0.19; Tables 2 and S1).

**Table 2:** Clinical measures collected before and during the pandemic

Measure	*n*	Pre-pandemic assessment		During-pandemic assessment		*t*	*p*	Cohen's *d*
		*M*	SD	*M*	SD			
MFQ-C	73	3.78	4.95	5.17	6.13	2.64	0.01*	0.31
MFQ-P	68	3.78	4.82	4.72	5.74	1.59	0.12	0.19
SCARED-C	73	15.18	13.33	18.52	16.12	3.09	0.003**	0.36
SCARED-P	69	15.33	13.25	16.85	13.37	2.33	0.02*	0.28

*SCARED-P* Screen for Child Anxiety Related Emotional Disorders-Parent, *SCARED-C* Screen for Child Anxiety Related Emotional Disorders-Child, *MFQ-C* Mood and Feelings Questionnaire-Child, *MFQ-P* Mood and Feelings Questionnaire-Parent

*p < 0.05, **p < 0.01, ***p < 0.001 uncorrected

From pre- to during-pandemic, 9.6% of participants crossed the clinically significant cut-off on youth-reported depression (MFQ-C), 11% on youth-reported anxiety (SCARED-C) and 11.6% on the parent-reported anxiety measure (SCARED-P).

### Pandemic-Related Stress and Worries as Mediators of Change

Changes in parent-reported anxiety (SCARED-P) were partially mediated by COVID worries (CRISIS-P) (*β* = 0.09, BCa CI [0.022, 0.192]), and pandemic stressors (CIS), (*β* = 0.10, BCa CI [0.013, 0.250]). Changes in youth-reported anxiety (SCARED-C) were mediated by COVID worries (CRISIS-P) (*β* = 0.10, BCa CI [0.026, 0.239]), but not pandemic stressors (CIS; *β* = 0.03, BCa CI [− 0.006, 0.163]). Changes in youth-reported depression (MFQ-C) were mediated by COVID worries (CRISIS-P) (*β* = 0.16, BCa CI [0.041, 0.365]), but not pandemic stressors (CIS; *β* = 0.06, BCa CI [− 0.001, 0.188]).

### Associations Between Pre-pandemic Threat Processing and Change in Anxiety

For the subsample with fMRI data, we found a trend increase in parent-reported anxiety during the pandemic (SCARED-P; *t*(45) = 1.96, *p* = 0.056, *d* = 0.29), but no increase in youth-reported anxiety (SCARED-C; *t*(44) = 1.32, *p* = 0.19, *d* = 0.20).

Neither attention bias (reaction time: incongruent—congruent trials) nor threat bias (threat—neutral trials) predicted during-pandemic anxiety for either informant (all *p*s > 0.08), controlling for pre-pandemic anxiety levels.

**Fig. 2:**
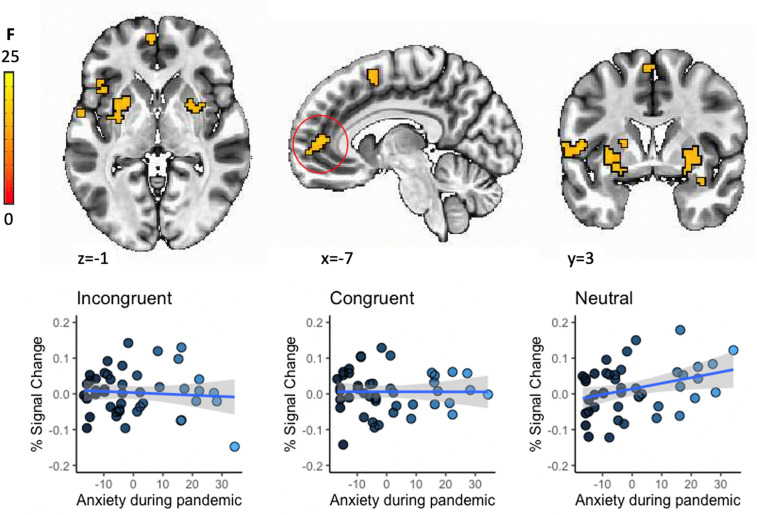
Regions showing significant condition-by-during-pandemic SCARED scores interaction at a whole-brain corrected threshold of *p* < 0.005. Post-hoc analyses of the anterior cingulate cluster showed a positive association between activation to neutral faces increases in anxiety during the pandemic, controlling for pre-pandemic anxiety levels

Brain activation in several prefrontal, parietal and subcortical clusters showed a significant association between pre-pandemic task activation and during the pandemic anxiety, controlling for pre-pandemic anxiety (SCARED-P; Table 3, Fig. 2). These interactions largely reflected positive associations between activation to neutral faces and during pandemic anxiety (all *Fs*(1.95,81.86) > 14.44, *ps* < 0.001). No findings emerged for the child-rated anxiety measure (SCARED-C).

**Table 3:** Summary of the results for the association between pre-pandemic brain activation to neutral and angry face conditions and during pandemic anxiety

Region	Cluster size		Coordinates (center of mass)			Coordinates (at peak)						Post-hocs: during-pandemic anxiety slope: r^a^
	k	mm^3^	CM LR	CM PA	CM IS	MI LR	MI PA	MI IS	Mean F	SEM	Max F	
R Putamen	175	2734	29.8	− 1	− 11.1	32.8	− 6.5	− 11.7	8.0437	0.1766	17.849	N: 0.003, *p* = 0.02
												IC: − 0.002, *p* = 0.03
												C: 0.001, *p* = 0.49
L Putamen	152	2375	− 27.2	2.5	1.2	− 30.3	3.3	0.7	7.3234	0.1195	17.338	N: .003, *p* = .01
												IC: − .001, *p* = = 0.34
												C: 0.002, *p* = 0.34
R Middle Frontal	116	1813	43.6	41.2	14	40.4	39.1	8	7.5735	0.1491	13.222	N: 0.004, *p* = 0.002
												IC: − 0.001, *p* = 0.82
												C: 0.000, *p* = 0.82
L Rolandic Operculum/Inferior Frontal Gyrus	110	1719	− 55.5	3	7.3	− 63.1	2.9	8.9	7.6134	0.1465	14.632	N: 0.001, *p* = 0.43
												IC: − 0.006, p < .001
												C: − 0.002, *p* = 0.26
L SMA	73	1141	− 1.4	− 1	64.4	5.1	− 7.4	62.7	7.0498	0.1351	11.372	N: 0.003, *p* = 0.14
												IC: − 0.002, *p* = 0.24
												C: 0.001, *p* = 0.34
L Middle Frontal Gyrus	62	969	− 36.6	36.3	35.8	− 40.4	27.4	37.4	7.1057	0.1699	11.019	N: 0.003, *p* = .05
												IC: − 0.002, *p* = .25
												C: 0.001, *p* = 0.44
L Inferior Frontal Gyrus	59	922	− 46.5	18.7	5.3	− 42.9	16.2	1.4	8.0479	0.2888	14.669	N: 0.002, *p* = 0.14
												IC: − 0.003, *p* = 0.007
												C: 0.001, *p* = .44
L Superior Medial Gyrus/Anterior Cingulate Cortex	55	859	− 5.6	54.9	6.4	− 2.5	57.4	3.6	8.3169	0.3233	15.902	N: 0.004, *p* = 0.04
												IC: − 0.001, *p* = 73
												C: − 0.003, *p* = 0.10
Inferior Frontal Gyrus/L Middle Orbital	49	766	− 26.2	34.1	− 15.9	− 25.3	30	− 18.5	7.6018	0.2544	13.758	N: 0.004, *p* = 0.009
												IC: − 0.002, *p* = 0.19
												C: 0.001, *p* = 0.44
L Middle Temporal Gyrus	48	750	− 66	− 31.3	6.2	− 70.7	− 35.7	6.8	7.6343	0.2478	13.295	N: 0.002, *p* = 0.18
												IC: − 0.004, *p* = 0.004
												C: 0.001, *p* = 0.59
L Inferior Parietal Lobule	48	750	− 35.7	− 43.5	52.1	− 35.4	− 45.5	49.8	7.3016	0.2466	13.336	N: 0.000, *p* = 0.83
												IC: − 0.004, *p* = 0.005
												C: − 0.002, *p* = 0.15

*Note*: Cluster-corrected voxel-wise multivariate model results are presented

*k* number of voxels in cluster, *mm3* cluster volume, *CM* center of mass of cluster, *MI* max intensity (peak), *SEM* standard error of the mean, *LR* left–right (x), *PA* posterior-anterior (y), *IS* inferior-superior (z), *N* Neutral, *IC* Incongruent, *C* Congruent

^a^Post-hoc correlations between during-pandemic anxiety and brain activation for each task condition after adjusting for age and pre-pandemic anxiety

## Discussion

The present study had three aims. First, we examined changes in anxiety and depression levels in youth with varying pre-existing psychopathology with the onset of the pandemic. Second, we directly assessed the role of pandemic-related stress and worries in change in these symptoms. Third, in a subsample, we examined whether biases in threat processing (behavioral and neural correlates) assessed pre-pandemic were related to pandemic-related changes in anxiety.

We found significant, albeit small, increases in anxiety (child- and parent-rated) and depression (child-rated) during the COVID-19 pandemic. Roughly 10% of children developed ‘clinically significant’ symptoms. Consistent with our hypotheses, change in routines and access to care as well as worries about infection partially mediated changes in anxiety and depression. These results confirm concerns about negative mental health consequences of the pandemic for youth [55], especially those who are exposed to more pandemic-related stressors [3, 22]. These results are in line with previous work detailing increases in internalizing symptoms in youth [3] and link these to pandemic-associated stress experiences [8, 17].

When studying pandemic-related stress, it is important to acknowledge that those stressors are multifaceted and family- and individual-specific [45]. Previous work has shown that populations experiencing the highest impact are often those with limited resources pre-pandemic with significant impact on basic needs such as access to food and medical care [45, 56, 57]. Participants in our sample had a median household gross income of $90,000—$179,999 and resided in Maryland or nearby states. While indicating that the pandemic significantly disrupted many aspects of daily life, most families indicated no impact on food access (~ 70%) and family income and employment (~ 65%). While only a small proportion of parents reported infection of self or family members (~ 10%), ~ 48% of parents reported a COVID-19 infection of member of their extended family. Thus, while the life and daily routines of families in our sample were changed dramatically, our sample may not represent those most profoundly affected by the pandemic.

Pre-pandemic activity of the middle frontal gyrus, anterior cingulate cortex, and bilateral putamen while viewing neutral faces was positively associated with anxiety during the pandemic. The few studies that have examined threat-related activation patterns assessed pre-pandemic as risk factors for increases in internalizing symptoms during the pandemic have also found hyperactivation in emotion generation and regulatory regions to be predictive of internalizing symptoms [37, 38]. Our results might indicate that hyper-activity of these regions, implicated in emotion regulatory [58] and reward [59] functions, during neutral face viewing renders individuals more vulnerable to anxiety. Previous work suggests that youth who are raised in environments with high uncertainty and threat (which places them at risk for later internalizing problems) tend to attribute hostile intent to ambiguous social cues including interpreting neutral faces as more threatening [60, 61]. Thus, heightened responses to emotionally ambiguous faces might reflect interpretation biases (i.e., neutral faces might be perceived as more threatening) [62] that are particularly impactful in the face of (pandemic-related) stress and uncertainty. Alternatively, in the context of angry faces, neutral faces could be associated with higher activity particularly in striatal regions because they are more rewarding, suggesting loss of reinforcers in the socially restrictive environment of the pandemic as an alternative mechanism. Given the modest sample size, it is important to consider these findings preliminary.

Several limitations need to be considered when interpreting the findings from the current report. First, while we used a rich multi-informant assessment of psychopathology, with both parent and child ratings, this multi-informant procedure also highlighted the lack of informant concordance across measures. Discordance between reporters is common in between child self-report and parent-report across internalizing and externalizing problems [47, 48]. While some differences in ratings may stem from measurement error, increasingly researchers acknowledge that each reporter may provide unique and valid information. Hence, where possible, it is important to collect both parent- and child-report, or ideally, rely on a clinician-reported measure. Our measures of pandemic related stress were based on parent-report, which may have impacted associations with symptom dimensions.

The COVID-19 pandemic is a constantly evolving stressor. We only collected data at a single time point during the pandemic with substantial temporal variance. With this heterogeneity in assessment, it is possible that some individuals crossed developmental periods (e.g., entered adolescence). Similarly, our pre-pandemic imaging and clinical measures were collected within two years prior to the pandemic. Ideally, families would have completed multiple assessments to capture the stability of associations and/or the dynamic unfolding of COVID-19 pandemic, although previous has shown no change in the association between anxiety and stress at two separate time points of the pandemic [22]. As schools and businesses reopen, it will be important to examine re-entry as its own potentially stressful event and assess the long-term consequences of the pandemic for youth development over the next years.

COVID-19 disruptions disproportionately burden youth and families from racial minority backgrounds, as well as those experiencing poverty [45]. Our sample was relatively affluent, which limits generalizability, another important limitation to consider when interpreting the results of the current study.

Some aspects of stress brought on by the pandemic are potentially unique and qualitatively different than previously studied community-wide disruptions (e.g., natural disasters). Specifically, containment and mitigation efforts such as social restrictions and quarantine result in a lack of in-person peer interactions, while other stressors (access to resources) appear in other contexts albeit exacerbated by the pandemic. Late childhood and adolescence is a time where youth navigate a larger social network outside of the family, with important implications for identity formation, independence, and social learning [13, 63]. Hence, examining long-term developmental effects of restricted social interactions is an important future direction for this work.

## Conclusion

The present work expands existing knowledge on the mediating role of psychological stress on symptoms of anxiety and depression in childhood and adolescence. It also provides preliminary evidence that enhanced brain activity in response to neutral faces renders youth more susceptible to the effects of psychological stress in terms of anxiety.

## Summary

The COVID-19 pandemic is a chronically stressful event, particularly impacting youth, and families. Global prevalence estimates of child and adolescent depression and anxiety reported to have doubled during the pandemic [10]. However, very few studies have used prospective longitudinal designs to examine how variability in clinical or neurocognitive factors assessed pre-pandemic influence youths’ stress response. Additionally, few studies have access to neuroimaging data, particular in clinically impaired youth, for whom psychological stressors may have a magnified impact. In this study, we leverage existing pre-pandemic well phenotyped clinical and imaging data in a transdiagnostic pediatric sample to examine (i) pandemic-related stress as a mediator of change in mood and anxiety symptoms, and (ii) threat processing biases as a predictor of increased anxiety during the pandemic. A clinically well-characterized sample of 81 youth including youth with affective and/or behavioral psychiatric diagnoses and without psychopathology completed two clinical assessments of symptoms, one before and another during the pandemic, and assessments of COVID-related worries and stress. A subsample also completed a threat processing fMRI task pre-pandemic. Results indicated that both anxiety and depression significantly increased during the pandemic. This symptom change was partially mediated by pandemic stress and worries. Additionally, in the subsample who completed the fMRI threat processing task, increased prefrontal brain activation in response to neutral faces pre-pandemic was associated with more intense anxiety during the pandemic. The present work extends existing knowledge on the mediating role of psychological stress on symptoms of anxiety and depression in vulnerable children and adolescents. It also provides preliminary evidence that enhanced brain activity in response to neutral faces renders youth more susceptible to experiencing increased anxiety during a stressful period.

### Supplementary Information

Below is the link to the electronic supplementary material.

Supplementary file1 (DOCX 157 kb)
