# Supplementary material for: Changes in Internalizing Symptoms During the COVID-19 Pandemic in a Transdiagnostic Sample of Youth: Exploring Mediators and Predictors
Source: Child Psychiatry Hum Dev. Author manuscript; Available in PMC 2024 Jan 6. (PMC9816351; doi:10.1007/s10578-022-01382-z)
Supplement: Supplement [file NIHMS1845977-supplement-Supplement.docx]

Supplementary Materials

**Changes in internalizing symptoms during the COVID-19 pandemic in a transdiagnostic sample of youth: Exploring mediators and predictors**

1. Image Preprocessing

A standard pipeline was applied, which included despiking, slice-timing correction, distortion correction, alignment of all volumes to a base volume (MIN_OUTLIER), non-linear registration to the MNI template, spatial smoothing to 6.5mm FWHM kernel (using blur_to_fwhm flag), masking, and intensity scaling. First-level models were created with generalized least squares time series fit with restricted maximum likelihood estimation of the temporal autocorrelation structure (3dREMLfit). This work utilized the computational resources of the NIH HPC Biowulf cluster (<http://hpc.nih.gov>). Both this processing and first-level general linear models (GLM) controlled for head motion. We censored any pair of successive TRs where the sum head displacement (Euclidean norm of the derivative of the translation and rotation parameters) between those TRs exceeded 1.0mm. TRs where more than 10% of voxels were flagged as outliers were also removed. Participants were excluded if the average motion per TR after censoring was greater than 0.25 mm or if more than 15% of all TRs were removed due to the either motion or outlier criteria (*n*=10 youth did not meet behavioral or motion cut-offs). Additionally, six head motion parameters (motion displacement along the x, y, and z axes as well as rotational movement [roll, pitch, and yaw]) were included as nuisance regressors in individual-level models. Regressors for three trial types of interest (congruent, incongruent, neutral) and error trials were included in first level GLMs. Events were modeled with a gamma hemodynamic response function.

1. fMRI threat processing task


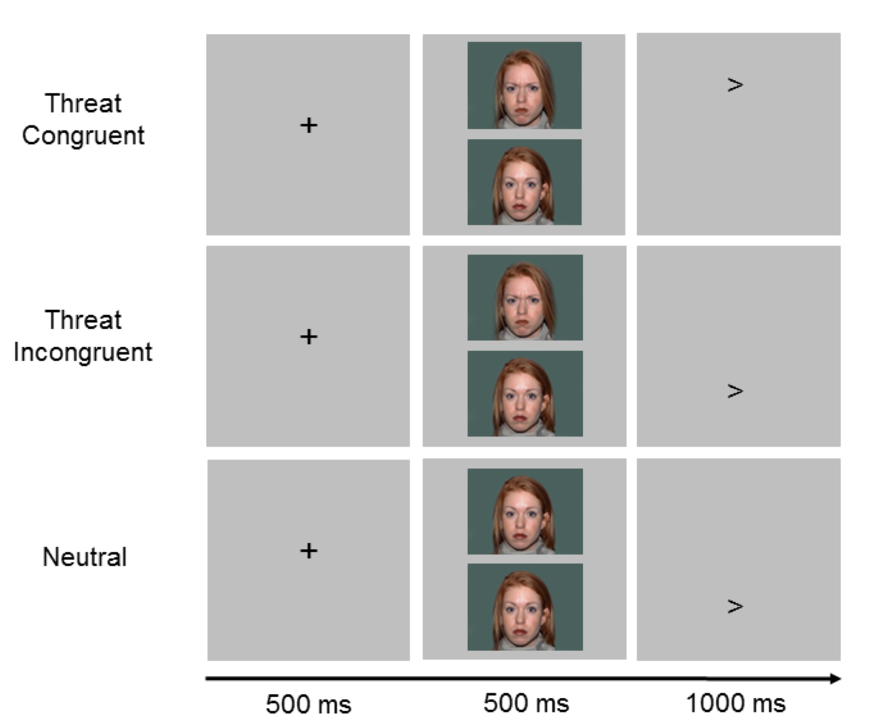


Figure S1. The task was provided by the Tel-Aviv University/NIMH Attention Bias Modification Treatment Initiative (http://people.socsci.tau.ac.il/mu/anxietytrauma/research/).

1. Additional analyses

To explore the role of pre-existing psychiatric diagnoses, we tested change in internalizing symptoms separately for youth with and without pre-existing diagnoses. Increases between youth with and without a pre-existing diagnosis were similar, if not larger in healthy controls, which is in line with other reports on youth with pre-existing internalizing psychopathology [1] or those at risk for internalizing psychopathology [2].

| **Measure** |  |  | **Pre-pandemic assessment** | | **During-pandemic assessment** | |  |  |  |
| --- | --- | --- | --- | --- | --- | --- | --- | --- | --- |
|  | ***Clinical status*** | ***n*** | ***M*** | **SD** | ***M*** | **SD** | ***t*** | ***p*** | **Cohen's *d*** |
| MFQ-C | HCs | 31 | 1.94 | 3.58 | 3.26 | 4.01 | 1.85 | .07 | .33 |
| MFQ-P | HCs | 27 | 1.93 | 2.84 | 2.96 | 4.86 | 1.51 | .14 | .29 |
| MFQ-C | Patients | 42 | 5.14 | 5.40 | 6.58 | 7.04 | 1.91 | .06 | .30 |
| MFQ-P | Patients | 42 | 4.99 | 5.46 | 5.85 | 6.03 | 1.02 | .31 | .16 |
| SCARED-C | HCs | 32 | 9.14 | 10.16 | 12.09 | 10.16 | 2.51 | .02* | .45 |
| SCARED-P | HCs | 28 | 8.39 | 10.91 | 10.89 | 10.71 | 2.23 | .03* | .42 |
| SCARED-C | Patients | 43 | 19.65 | 13.73 | 23.31 | 17.73 | 2.19 | .04* | .34 |
| SCARED-P | Patients | 44 | 19.85 | 12.77 | 20.96 | 13.58 | 1.41 | .17 | .22 |

Note. SCARED-P: Screen for Child Anxiety Related Emotional Disorders-Parent, SCARED-C: Screen for Child Anxiety Related Emotional Disorders-Child, MFQ-C: Mood and Feelings Questionnaire-Child, MFQ-P: Mood and Feelings Questionnaire-Parent.

HC: Healthy Controls

*p < .05, **p < .01, ***p < .001 uncorrected.

*Table S2.* Associations between clinical and stress measures.

|  | **1** | **2** | **3** | **4** | **5** | **6** | **7** | **8** | **9** | **10** | **11** |
| --- | --- | --- | --- | --- | --- | --- | --- | --- | --- | --- | --- |
| 1. Pre-Pandemic MFQ-C |  |  |  |  |  |  |  |  |  |  |  |
| 2. Pre-Pandemic MFQ-P | 0.53*** | |  |  |  |  |  |  |  |  |  |
| 3. Pre-Pandemic SCARED-C | 0.69*** | 0.28* |  |  |  |  |  |  |  |  |  |
| 4. Pre-Pandemic SCARED-P | 0.41*** | 0.61*** | 0.51*** |  |  |  |  |  |  |  |  |
| 5. During-Pandemic MFQ-C | 0.69*** | 0.24 | 0.72*** | 0.37** |  |  |  |  |  |  |  |
| 6. During-Pandemic MFQ-P | 0.53*** | 0.54*** | 0.35** | 0.56*** | 0.55*** |  |  |  |  |  |  |
| 7. During-Pandemic SCARED-C | 0.56*** | 0.21 | 0.81*** | 0.56*** | 0.84*** | 0.46*** |  |  |  |  |  |
| 8. During-Pandemic SCARED-P | 0.50*** | 0.59*** | 0.46*** | 0.78*** | 0.55*** | 0.71*** | 0.59*** |  |  |  |  |
| 9. CIS | 0.19 | 0.49*** | 0.19 | 0.44*** | 0.30* | 0.57*** | 0.23 | 0.50*** | |  |  |
| 10. CRISIS-P COVID-19 Worries Subscale | 0.42*** | 0.24 | 0.38** | 0.38** | 0.55*** | 0.57*** | 0.45*** | 0.50*** | 0.53*** |  |  |
| 11. Age | 0.07 | -0.07 | 0.02 | -0.21 | 0.07 | -0.15 | -0.05 | -0.18 | -0.07 | -0.09 |  |
| 12. Income | -0.11 | -0.04 | 0.09 | 0.04 | -0.14 | -0.08 | 0.08 | 0.01 | -0.13 | -0.26 | -0.23 |

Note. SCARED-P: Screen for Child Anxiety Related Emotional Disorders-Parent, SCARED-C: Screen for Child Anxiety Related Emotional Disorders-Child, MFQ-C: Mood and Feelings Questionnaire-Child, MFQ-P: Mood and Feelings Questionnaire-Parent, CIS: Coronavirus Impact Scale, CRISIS-P: Coronavirus Health Impact Survey - Parent. *p < .05, **p < .01, ***p < .001uncorrected.

**References**

1. Sadeghi, N., et al., *Mood and Behaviors of Adolescents With Depression in a Longitudinal Study Before and During the COVID-19 Pandemic.* Journal of the American Academy of Child & Adolescent Psychiatry, 2022.

2. Morales, S., et al., *Neurocognitive Profiles in Adolescence Predict Subsequent Anxiety Trajectories during the COVID-19 Pandemic.* Biological Psychiatry: Cognitive Neuroscience and Neuroimaging, 2021.
